# Diseases Detection of Occlusion and Overlapping Tomato Leaves Based on Deep Learning

**DOI:** 10.3389/fpls.2021.792244

**Published:** 2021-12-10

**Authors:** Xuewei Wang, Jun Liu, Guoxu Liu

**Affiliations:** ^1^Shandong Provincial University Laboratory for Protected Horticulture, Blockchain Laboratory of Agricultural Vegetables, Weifang University of Science and Technology, Weifang, China; ^2^College of Information and Control Engineering, Weifang University, Weifang, China

**Keywords:** YOLOv3-tiny, inverse-residual block, field images, multi-scale, occlusion and overlapping, robust

## Abstract

**Background:** In view of the existence of light shadow, branches occlusion, and leaves overlapping conditions in the real natural environment, problems such as slow detection speed, low detection accuracy, high missed detection rate, and poor robustness in plant diseases and pests detection technology arise.

**Results:** Based on YOLOv3-tiny network architecture, to reduce layer-by-layer loss of information during network transmission, and to learn from the idea of inverse-residual block, this study proposes a YOLOv3-tiny-IRB algorithm to optimize its feature extraction network, improve the gradient disappearance phenomenon during network deepening, avoid feature information loss, and realize network multilayer feature multiplexing and fusion. The network is trained by the methods of expanding datasets and multiscale strategies to obtain the optimal weight model.

**Conclusion:** The experimental results show that when the method is tested on the self-built tomato diseases and pests dataset, and while ensuring the detection speed (206 frame rate per second), the mean Average precision (mAP) under three conditions: (a) deep separation, (b) debris occlusion, and (c) leaves overlapping are 98.3, 92.1, and 90.2%, respectively. Compared with the current mainstream object detection methods, the proposed method improves the detection accuracy of tomato diseases and pests under conditions of occlusion and overlapping in real natural environment.

## Introduction

Tomato is one of the most popular crops planted in China, and it has an irreplaceable position in vegetables, fruits, medicinal, and other aspects, with a huge planting volume and demand (Li, 2012). Taking Shouguang City, Shandong Province as an example, Shouguang City’s tomatoes are mainly produced in Luocheng Street, with about 12,000 household greenhouse planters and 20,000 winter-warm greenhouses, with an annual trading volume of 360 million kilograms, and an annual trading volume of 730 million yuan. The products are exported to Russia, North Korea, Myanmar, and other countries. This town is an important tomato production and sales base in Shandong Province and enjoys the reputation of “small town with tomato characteristics.” According to statistics, a common 100-m greenhouse has a revenue of at least RMB 100,000, and vegetable farmers have realized the “income-increasing dream” through tomatoes.

Traditionally speaking, tomatoes belong to seasonal fruits and vegetables, but the market has a great demand for tomatoes in each season. To meet the market demand and improve economic benefits, most farmers use greenhouse planting to overcome the influence of season, temperature, and other environmental factors, and achieve tomato planting and production for more than three seasons in 1 year. From the previous field research and feedback from farmers, we know that the whole growth cycle of tomatoes has strict requirements on the growth environment, planting methods, pest control, and other aspects, and the requirements on the external environment of tomatoes at all growth stages of the whole growth cycle are also of high standards and are different. In recent years, the impact of diseases and pests on tomato cultivation has been aggravated. The main reason is that the optimized planting structure and complete water and fertilizer supply conditions are not only conducive for tomato growth, but also provide convenience for the occurrence of diseases and pests. At the same time, the unscientific and non-standard use of pesticides also cause the increasing resistance of pathogens. Also, the differences of tomato varieties and cross-hazards are the causes of the growing severity of tomato diseases and pests (Wang et al., 2018). Therefore, the cost, time, and labor consumption of high-quality tomato cultivation are relatively high. However, most of the peasant households have not received professional knowledge, and they do not know the symptoms of diseases, pests, and other causative factors.

During tomato planting, the information of diseases and pests, the demand of crop growth environment, and the control measures mostly depend on the communication between peasant households and the previous planting experience. It is difficult to grasp the diseases and pests that may occur in a certain planting stage under certain conditions. It is also difficult to accurately determine the types of diseases and pests and their control methods. These practical problems have a great impact on tomato production.

The investigation revealed that the major diseases of tomato included 34 infestation diseases and 39 physiological diseases, and the disease characteristics were mainly focused on the color and morphology of the lesions. To make the research typical and better feasible, in this work, 12 common diseases including early blight, late blight, yellow leaf curl virus, gray leaf, coal pollution, gray mold, leaf mold, navel rot, leaf curl disease, mosaic, leaf miner, and greenhouse whitefly were selected for research.

The traditional method of identifying tomato diseases and pests is usually manual identification, that is, growers make subjective judgment based on planting experience or text data, or image comparison through the network, books, etc., or ask pathologists to analyze and identify tomato diseases and pests. Traditional manual diseases and pests identification takes a lot of time and effort, and is often accompanied by very high subjectivity. Subjective evaluation is susceptible to personal factors and external factors (such as light, occlusion, and overlapping). It is inefficient and has large errors, which can easily lead to the wrong diagnosis and wrong medication of tomato diseases and pests. Severe conditions can also cause pollution to water sources, soil, and so on. In addition, due to the scattered agricultural production in China and the lack of relevant agricultural experts, there are some limitations in the support provided by tomato pathologists in the professional pathological analysis and decision methods. Therefore, the manual identification of diseases and pests cannot meet the requirements of high-efficiency tomato production in the development of modern agriculture, an so the automatic and accurate detection of tomato diseases and pests is urgently needed.

Traditional plant disease detection relies on a large amount of manual design, where the model generalization performance is poor and the detection accuracy cannot meet the practical demand. Thanks to the rapid development of deep learning, Girshick et al. (2013) proposed that Region-CNN (R-CNN) and the precision of detection was substantially improved. Subsequent researchers have made improvements from a number of perspectives based on R-CNN. Fast R-CNN (Girshick, 2015) was proposed, and the detection efficiency is improved by sharing the multitask loss function and convolution weights. Faster R-CNN (Ren et al., 2017) integrates region nominations with convolutional neural networks and truly implements an end-to-end target detection framework. Mask R-CNN (He et al., 2017) introduced region of interest (ROI) align to replace ROI pooling and enable segmentation and detection of images. Region-based Fully Convolutional Networks (R-FCN) (Dai et al., 2016) introduced fully convolutional operation and the detection effect is improved greatly. FPN (Lin et al., 2017) and CascadeR-CNN (Cai and Vasconcelos, 2017) have achieved an extremely high detection accuracy and approximate the resolving power of humans. The above detection framework all contain both regional nominations and detection networks, and they are called two-stage methods. Other researchers have proposed region free nomination stage that unifies classification and detection tasks, and they are called one-stage methods. For example, YOLO (Redmon et al., 2016), RetinaNet (Lin et al., 2018), RefineDet (Zhang et al., 2017) are typical one-stage methods, and the real-time performance is greatly improved.

With the deep integration of modern information technology such as Internet of Things, Cloud Computing, and Artificial Intelligence with agriculture, smart agriculture has become a major trend in the development of modern agriculture in the world through the implementation of whole industry supply chain with real-time information perception, quantitative decision-making, intelligent production control, and precise personality management, and has made important progress in the field of crop harvesting (Xu et al., 2020). In the field of crop diseases and pests identification, Intelligent Agriculture relies on the Internet of Things system built by fixed monitoring cameras, mobile equipment, robots, smartphones, and other terminals. The classification and detection of network based on deep learning method is studied on the basis of real-time collection and acquisition of a large number of high-quality image dataset of crop diseases and pests, which can provide accurate, low-cost, high-efficiency, reliable, and real-time results for broad agricultural producers. It has gradually become the focus of research at home and abroad. Computer vision provides a very effective means for automatic detection of crop diseases and pests, and some progress has been made (Ouhami et al., 2020). Under natural scenes, the tomato diseases and pest objects are often covered by light and shade, and the branches and leaves are covered or in an overlapping state. The identification and localization of the tomato diseases and pests objects under the influence of shading and overlapping is a difficult problem that must be solved.

**FIGURE 1:**
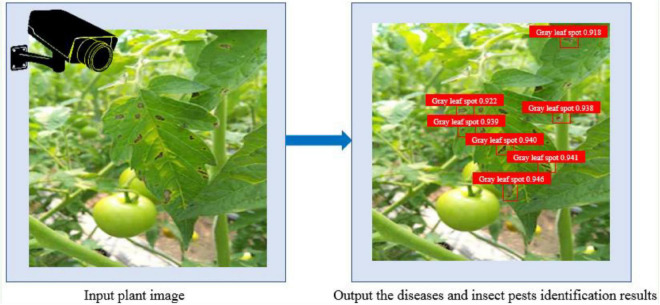
An example illustrating plant diseases and pests identification.

With the rapid development of smart agriculture, the technology of using cameras to determine whether plants appear in images are infected with diseases and pests has been applied in the field of smart agriculture, which plays an increasingly important role in plant protection. This technique of using computer vision and machine learning to determine whether a particular plant in a camera is affected by diseases or pests is called plant diseases and pests identification, as shown in Figure 1.

Plant diseases and pests identification not only has a very urgent application need, but also has a very important research value. In recent years, plant diseases and pest identification has attracted wide attention from academia and agriculture, and become a research hotspot in the field of computer vision. After more than 10 years of development, a large number of plant diseases and pests identification models have been proposed at home and abroad, and very high accuracy has been achieved under the limited simulation conditions (Singh et al., 2018; Geetharamani and Arun Pandian, 2019; Shekhawat and Sinha, 2020), and even surpasses the ability of human vision.

In recent years, some progress has been made in the research of plant diseases and pest identification under natural scenes. Fuentes et al. (2019) proposed an improved Faster R-CNN algorithm, which can effectively detect and locate plant abnormalities. The average accuracy of 92.5% was achieved in the built tomato plant abnormality description dataset. However, the real-time performance of the algorithm was not strong. Anagnostis et al. (2020) took images of walnut anthrax in orchards under various light conditions. A total of 4,491 images of leaves with and without anthrax were collected. The images of leaves infected with anthrax reached 2,356, slightly more than the images of healthy leaves. The classification accuracy of walnut anthrax was as high as 98.719% using convolutional neural network. Prabhakar et al. (2020) used ResNet101 to measure the severity of early blight of tomato leaves and the accuracy reached 94.6%. But their methods can only judge whether the disease was infected or not and cannot locate the disease. They mainly aim at the target recognition of a small number of images taken in close range, which is difficult to apply in practice. Zhao and Qu (2019a,b) used YOLOv2 algorithm to detect healthy and diseased tomatoes, and the mean Average precision (mAP) was as high as 91%. However, the method did not take into account the small and dense multiobject occlusion and overlap problem in natural environment. Liu and Wang (2020a,b) proposed an improved YOLO V3 algorithm for tomato diseases and pests detection with high accuracy and speed, but this method can only effectively detect tomato diseases and pests targets in the case of slight leaf overlap, and there is no satisfactory detection result in the case of large area occlusion. There are often uncertainty issues such as posture, background, and occlusion of leaves in the detection of plant diseases, which can greatly affect the detection accuracy. So, increasing the object detection accuracy has always been paid great attention to Liu et al. (2020) proposed an improved tomato detection model based on YOLOv3 aiming at complex environmental conditions, such as light change, branching, leaf blockage, and tomato overlap, which integrated a dense architecture for feature reuse, but the model was only used for tomato fruit positioning and could not be used for tomato diseases and pests detection. All the above literatures utilize the excellent learning ability, flexibility, and adaptability of convolutional neural network to solve the problems of time-consuming, laborious, and low accuracy in plant diseases and pests detection under complex background. However, in the above studies, the leaves of plant diseases and pests are mostly sparse and complete, and the characteristics of diseases and pests are obvious. In this work, the images of tomato diseases and pests are collected under different light conditions in the real natural environment, and there are even sunlight shadows or sundries, such as branches and trunks, or the leaves overlap densely. These factors are obstacles in the detection of tomato diseases and pests. To effectively carry out real-time detection for multiple objects, an improved object detection model based on YOLOv3 needs to be proposed for issues such as small objects and occluded objects prone to being missed or inaccurate detection frame positioning.

In the real natural environment, the study of plant diseases and pests identification has its particularity. In the real agricultural Internet of Things video monitoring, there are various shooting equipments, the image quality of plant diseases and pests objects is poor, the resolution is low, and there are also obvious changes in perspective and light (Barbedo, 2018). Therefore, compared with general image recognition, plant diseases and pests identification still faces the following problems: (1) In different monitoring and shooting equipment, the distance between plants and shooting equipment is different, resulting in different resolution, light, and perspective of plant diseases and pests image under different shooting equipment horizons, and different visual characteristics of the same plant diseases and pests image will produce obvious changes; (2) Different degree of occlusion caused by background and other factors leads to a large number of occlusion problems, which lead to poor identification of plant diseases and pests; (3) Due to the changes of leaf posture and shooting equipment angle, the differences of visual characteristics between different images of plant diseases and pests may be small in different shooting devices. In addition, some specific problems have not been paid enough attention to. For example, large-scale and fast retrieval problems, insufficient data problems, complex and crossmodal problems of plant diseases and pests occurrence in the actual agricultural environment, make the problem of plant diseases and pests identification more difficult than the general case-based image retrieval.

Investigation on the field environment of tomato greenhouse base showed that tomato plants grew densely, and light shading, branch and leaf occlusion, and overlap accounted for about 21.2%. Thus, tomato diseases and pests detection under conditions of occlusion and overlapping become the key and difficult point of the research. To solve the problem of rapid and high-precision detection of tomato diseases and pests in real natural environment, this work proposes a method to enhance the learning of foreground region features by occluding overlapping object foreground region samples, chooses YOLOv3-Tiny model based on regression method, and proposes a YOLOv3-tiny-IRB network structure with inverse residual blocks. Depth-wise convolution is used to reduce the model parameters, and an inverse residual module is constructed to extract high-dimensional features, and a linear activation function is used to reduce the loss of information caused by the channel combination process. The improved object detection network is trained by fusing data amplification and multiscale training strategies. The detection effect of the method in this study is significantly improved under two kinds of interference scenarios, i.e., sundries occlusion and blade overlap.

## Experimental Data

### Dataset Acquisition

The experimental tomato planting base is located in Shouguang City, Shandong Province. By Using smartphone, digital camera, and other monitoring equipment with various resolutions, 15,000 images of early tomato diseases and pests during the growth and development period were collected. The weather during image acquisition includes sunny and cloudy days, and the acquisition period is 8:00-18:00, which covers possible lighting conditions such as sunshine, backlight, and sidelight. Greenhouse tomato leaves are photographed in multiple orientations so that the main features of the diseases and pests can be shot, such as texture, color, shape, etc. Each image is formatted as JPG. Images were cropped to 224 × 224 pixel size.

Five thousand images containing the following three representative scenarios were screened from 15,000 tomato diseases and pests images.

(a) Leaves sparse and complete. The objects are relatively clear and easy to identify.
(b) Branches or sunlight shade or other debris occlusion. It is possible that there are situations in which diseases and pests are too small, adherent, mutually obscured, or obscured by the shoot and leaves, increasing the difficulty of detection.
(c) Leaves overlapping densely. Overlapping bounding boxes may be erroneously discarded, leading to missed observations with a larger probability.

The number of each species of diseases and pests is shown in Table 1.

**TABLE 1:** The number of each species of diseases and pests.

Species	Number
Early blight	401
Late blight	416
Gray leaf spot	425
Brown spot	431
Coal pollution	408
Gray mold	421
Leaf mold	419
Powdery mildew	402
Leaf curl	418
Mosaic	413
Leaf miner	411
Greenhouse whitefly	435
Total	5000

From 5,000 representative tomato diseases and pests images, 3,500 were randomly selected as original training images (containing 21,038 tomato diseases and pests objects), and the remaining 1,500 were selected as test images (containing 9,067 tomato diseases and pests objects).

### Image Enhancement

The image enhancement of training samples can improve the quality and diversity of samples, which is conducive to the improvement of CNN detection accuracy (Ding and Taylor, 2016). Under natural light of greenhouse planting base, especially when the light is very strong, due to the mutual occlusion of tomato plant leaves or backlight photography, the leaf surface produces shadows, which makes the image characteristics of tomato diseases and pests very different from those under normal light. Especially, some relatively small objects are not obvious in the image, affecting the quality of tomato diseases and pests image. The quality of training samples can affect the detection effect of the model, and so the contrast of the image needs to be adjusted to improve the detection effect of the detection model. In this study, adaptive histogram equalization (Algorithms, 2015) was used to enhance tomato diseases and pests images, improve the gray dynamic range of images, effectively improve the contrast of images, and enrich the details of images, which is equivalent to adjusting the image brightness and reducing the impact of light on image quality.

### Sample Labeling

**FIGURE 2:**
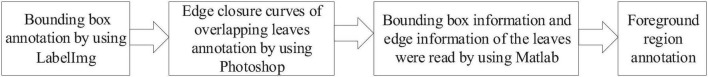
The process of sample labeling.

To improve the detection accuracy of tomato diseases and pests, various appearances and shapes of the objects were fully considered in the sample labeling process in this study. Manual labeling, interactive labeling, and Matlab programming were used for labeling. The process of sample labeling is shown in Figure 2.

(1) LabelImg, an open source annotation tool, was used to annotate the bounding boxes of 21,038 tomato diseases and pests objects in 3,500 original training images (no annotation was made when the object was covered by more than 70% of the area). Using this software, images in the dataset can be annotated as *.xml and *.txt files. The annotated file saves information such as class, size, and location of each object in the image. Also, LabelImg (TzuTa, 2017) was used to annotate 9,067 tomato diseases and pests object bounding box in 1,500 test images (no annotation was made when the object was covered by more than 70%). Considering that the test dataset is used to evaluate the detection accuracy of the model, the test dataset does not need to mark the object foreground area.

(2) The edge closure curves of overlapping leaves were automatically generated by using the quick selection tool of Photoshop software. However, for leaves with uneven surface color and illumination, it is difficult to automatically generate accurate edges, and the edge contours of leaves need to be manually marked.

(3) Using Matlab programming, the bounding box information and edge information of the leaves were read, and the pixels of the area outside the edge contour curve in the bounding box of the object were set to 0.

(4) In view of the difficulties caused by occlusion or overlap in tomato diseases and pests detection, a method of enhancing the learning of convolutional features of tomato diseases and pests foreground regions by annotating the foreground regions of training samples is proposed. Firstly, by manual annotation method, the pixels of the object background area were set to zero to obtain the object foreground area samples, and the object foreground area samples were trained to reduce the interference of non-foreground features in the bounding box, so as to enhance the learning of foreground features by the network and obtain the tomato diseases and pests detection network. When labeling samples, the pixels of the object background area in the labeling bounding box were set to 0, whereas the pixels of the foreground area remain unchanged. Thus, when convolution feature extraction is performed, the influence of unrelated information features on the feature extraction of tomato diseases and pests can be reduced. At the same time, to retain the color, shape, and texture of the edge of tomato diseases and pests, the object foreground area in the bounding box included a certain area (5–10 pixels) around the object contour to enhance the model’s learning of the characteristics of the foreground area including, the object edge.

### Data Amplification

The training sample was expanded in this study. Considering that most of the tomato diseases and pests on the leaves of the tomato plant are naturally suspended, while some of them are inclined at multiple angles due to the occlusion of branches or other leaves, this study conducted horizontal mirror inversion and rotation operations on the training samples. The rotated image is intercepted in the center. After rotating, the object near the edge in the image will be discarded if it is incomplete or completely lost.

### Dataset Preparation

**FIGURE 3:**
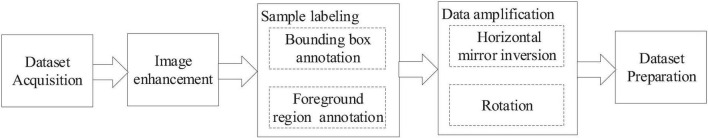
The process of dataset preparation.

The process of dataset preparation is shown in Figure 3.

Datasets and sample size are shown is Table 2. In datasets A and B, the number of bounding boxes for tomato diseases and pests was 21,038, and the target foreground area (2,987) was marked for occlusion and overlapping tomato diseases and pests leaves. In dataset C, the number of annotations for the bounding box of tomato diseases and pests was 1,73,304, and the number of annotations for the foreground area was 24,158.

**TABLE 2:** Datasets and sample size.

Datasets	Data processing method	Sample size	Number of annotation	
			Bounding box annotation	Foreground area annotation
A	No	3500	21038	2987
B	Image enhancement	3500	21038	2987
C	Data amplification	29016	173304	24158

Considering that the test set is used to evaluate the detection accuracy of the model, the original image annotated by the bounding box is used as the test set.

## Method of Improving YOLOv3-Tiny

### Principle of YOLOv3-Tiny

YOLO detection (Redmon et al., 2015) has developed three generations, and many networks for specific scenes have been derived. YOLO first uses the idea of regression to classify image objects, and the detection speed reaches 45 frames/s. The disadvantage is that the detection accuracy of small objects is not high. YOLOv2 (Redmon and Farhadi, 2017) have optimized the model structure of YOLO and improved the detection speed, but the detection accuracy was not improved. YOLOv3 (Redmon and Farhadi, 2018) uses deep residual network to extract image features, as the minimum feature map for feature extraction is too large, the detection speed is reduced and the detection effect for medium or large size objects is not good.

YOLOv3-tiny (Redmon, 2018) compresses the original network version without residual layer, and only two YOLO output layers with different scales are used, which improves the detection speed and accuracy of small object detection. Since tomato diseases and pests image objects are mostly small objects, and the detection speed requirements are high, it is suitable for the basic network of this detection. It uses end-to-end object detection, while ensuring accuracy, and it can greatly improve the detection speed.

### Existing Problems of YOLOv3-Tiny

In the feature extraction process of YOLOv3-Tiny model, the number of network layers in the backbone network is small, the extracted feature information is less effective, and the extraction effect is poor. Therefore, each region in the extracted feature map should be given different weights to better perform classification task. In addition, the original model cannot make full use of the feature information output from the shallow layer of the network, resulting in poor fine-grained detection ability of the model.

### The Improved YOLOv3-Tiny Network (YOLOv3-Tiny-IRB)

**FIGURE 4:**
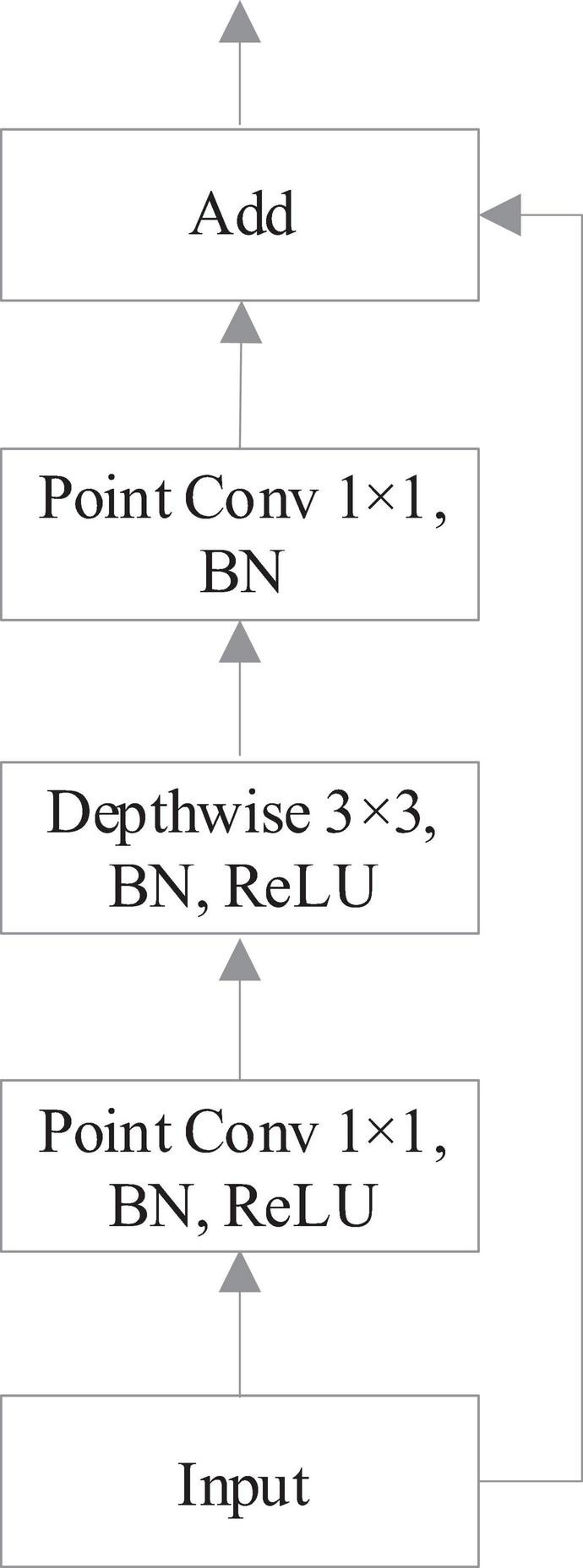
The structure of the inverse-residual block.

In view of the above problems, this work improves the original network and optimizes YOLOv3-Tiny to make it more suitable for tomato diseases and pests object detection task based on field images with multiscale occlusion. In order to solve the problem that the storage and computation of conventional convolution parameters multiply with the deepening of network layers, resulting in the increase of model size and difficult application in hardware platforms with limited computing resources, this work introduces the idea of residual blocks in Resnet (He et al., 2016). Instead of conventional convolution, depth-wise separable convolution is applied to construct inverse-residual block, which transforms the “spatial cross-channel” features learning process into two parts: spatial feature learning and channel combination. Specifically, one is that depth-wise separable convolution performs spatial convolution independently on each input channel; the other is that point where convolution maps the output results of depth-wise separable convolution to a new channel space. The structure of the inverse-residual block is shown in Figure 4.

According to Figure 4, firstly, the input step size Stride = 1, the number of channels is adjusted by 1 × 1 convolution kernel, and the results are obtained by batch normalization (BN) algorithm and rectified linear unit (ReLU) activation function in turn; secondly, the network features are extracted by 3 × 3 convolution kernel, and pass through the ReLU function of BN algorithm; thirdly, the number of channels is adjusted by 1 × 1 convolution kernel to get the output through BN algorithm. Finally the output is added to the input before entering the structure. The structure of inverse-residual block is different from the residual in ResNet. ResNet first reduces dimension, then convolutes, and finally increases dimension, whereas inverse-residual block first increases dimension, then convolutes, and finally reduces dimension. The 1 × 1 convolution dimension enhancement is used to increase the expressive ability of the model. When the channel information is processed with the ReLU function, the channel will inevitably lose information. When there are enough channels, the lost information of one channel may still remain in other channels, so it is necessary to increase the dimension of the features first. The input of the inverse-residual block structure already contains all of the necessary information, so the ReLU activation layer is not added after the final 1 × 1 convolution to prevent information loss. After the dimension is increased, the information is more abundant. At this time, the ReLU function is added to increase the sparsity of the network. After dimension reduction, the necessary information can be maintained without loss.

The calculation of inverse-residual block in this study is shown in Table 3.

**TABLE 3:** Inverse-residual block parameters.

Input	Operation	Output
*h* × *w* × *k*	1 × 1 point conv, ReLU	*h* × *w* × 2*k*
*h* × *w* × 2*k*	3 × 3/s depthwise conv, ReLU	*h*/*s* × *w*/*s* × 2*k*
*h*/*s* × *w*/*s* × 2*k*	1 × 1 point conv, Linear	*h*/*s* × *w*/*s*×2*k*

In Table 3, *h* and *w* are the height and width of the feature map, respectively, *k* is the number of channels of the feature map, *t* is the multiple of the number of expanded channels, and *s* is the step size. According to Table 2, both point convolution and depth-wise convolution of the extended channel in the inverse-residual structure of this study apply ReLU non-linear activation function. When the point convolution layer for the number of combined channels uses ReLU activation function, the negative values will be changed to 0, thus losing part of the information, and the linear activation function is used to solve the information loss problem in the process of combined channels.

**FIGURE 5:**
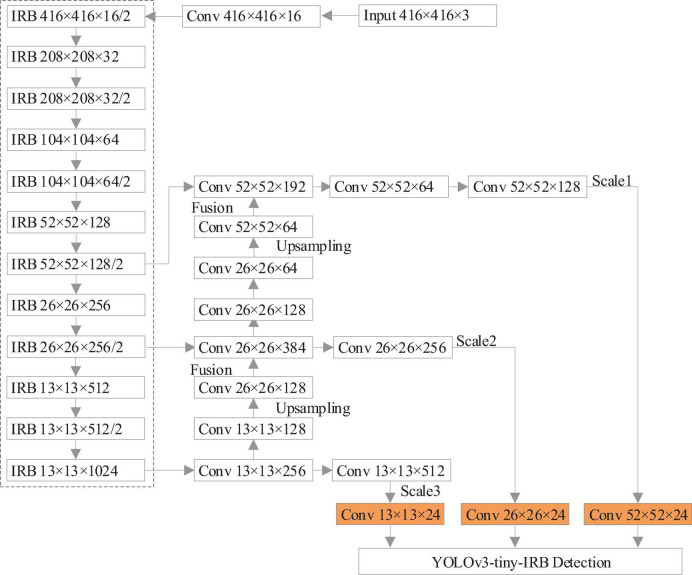
The improved YOLOv3-tiny network model (YOLOv3-tiny-IRB).

The improved YOLOv3-tiny network is denoted as YOLOv3-tiny-IRB, and the network structure is shown in Figure 5, where the IRB (Sandler et al., 2018) is an Inverse Residual Block and the dotted box is the part of network feature extraction.

According to Figure 5, in the feature extraction network, the feature extraction quantity is improved by increasing the convolution layer, and the convolution with step size of 2 is used to replace the maximum pooling layer in the original network for downsampling. The inverse residual block constructed by depth-wise separable convolution is used instead of traditional convolution. The improved network is composed of 12 inverse-residual blocks, which extract high-dimensional features through inverse-residual blocks, expand feature map channels, and then carry out channel dimension reduction to obtain feature maps to make up for the deficiency of the algorithm in occlusion object detection and improve the accuracy of the algorithm. While increasing feature extraction, the model size and parameter calculation amount are effectively reduced. At the same time, there is downward transmission among scales, and the scale diversity caused by different degree of occlusion and depth of visual field decides to add an upper sampling layer on the basis of the two-scale prediction objects of the original network, which forms a three-scale prediction of 52 × 52, 26 × 26, 13 × 13. Fusion of different size features is conducive to the different object sizes in occlusion scenarios, preventing overfitting and further improving the accuracy of object detection.

Table 4 lists the size and computation amount required to process an image of YOLOv3, YOLOv3-tiny, and the network model improved in this work. It can be seen that the network model improved in this study is only 0.5M larger than YOLOv3-tiny, and the amount of computation required to process an image increases by 0.24GFLOPs, which is much smaller than that of YOLOv3 model. It has great advantages in model size and calculation amount. It meets the real-time detection requirements of the embedded system.

**TABLE 4:** Size and computation amount of different network models.

Network models	Model size/M	Floating point calculation amount/GFLOPs
YOLOv3	246.5	65.7
YOLOv3-tiny	34.7	5.56
YOLOv3-tiny-IRB	35.2	5.80

### Anchor Parameter Optimization

When studying object detection, appropriate anchor value can improve the detection accuracy and speed. The anchor value in the original YOLO algorithm is calculated by K-means clustering method, which is more accurate than manual calculation. However, for the dataset of tomato diseases and pests in this work, the anchor value obtained by the original algorithm using COCO and VOC datasets with too large instance size is too large, so it is necessary to recalculate the appropriate anchor value according to the actual data. In tomato diseases and pests detection, clustering is to maximize the IOU value of the ratio of anchor box to ground truth, so IOU is used as the objective function to determine the distance, and its formula is as follows:


(1) $$d\left(box,centroid\right)=1-IOU_{centroid}^{box}$$

Therefore, we set the center of centroid as the cluster center in each instance label, and BOX as the bounding box. The smaller the IOU, the larger the distance.

According to the label information of all the examples in the study, new anchor values are obtained, which are: (10, 12), (22, 24), (30, 32), (69, 86), (83, 105), (119, 192), (168, 264), (223, 296), (311, 358). Three groups of smaller anchor boxes are assigned to larger size feature maps for predictive use; three groups of middle size anchor boxes are assigned to medium size feature maps for predictive use. In addition, three groups of anchor boxes with larger area are allocated to smaller size feature map prediction.

**FIGURE 6:**
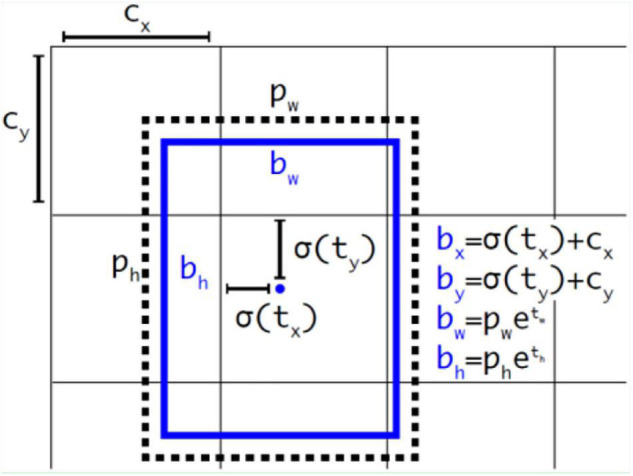
Bounding box prediction.

Each grid uses the method of directly predicting relative position to calculate three prediction boxes, as shown in Figure 6.

The relevant formulas in Figure 6 are as follows:


(2) $$b_{x}=\sigma \left(t_{x}\right)+c_{x}$$

(3) $$b_{y}=\sigma \left(t_{y}\right)+c_{y}$$

(4) $$b_{w}=p_{w}e^{t_{w}}$$

(5) $$b_{h}=p_{h}e^{t_{h}}$$

In the above-mentioned formulas, *c*_*x*_ and *c*_*y*_ represents the upper-left coordinates of each grid. Here, *p*_*w*_ and *p*_*h*_ represent the width and height of mapping from the anchor to the feature map, respectively, and *t*_*x*_, *t*_*y*_, *t*_*w*_, *t*_*h*_ are the goals of model learning.

## Network Training

### Experimental Running Environment

The experimental hardware environment of this study is shown in Table 5. On this basis, the software environment is built: Ubuntu 16.04, Python, OPPENCV, CUDA, etc. The framework uses Caffe and darknet-53 framework.

**TABLE 5:** Experimental hardware environment configuration.

Hardware name	Model	Number
Main board	ASUS WS X299 SAGE	1
CPU	INTEL I7-9800X	1
Memory	Kingston 16G DDR4	2
Graphics card	GEFORCE GTX1080Ti	2
Solid-state hard disk	Kingston 256G	1
Hard Disk	Western Number 1T	1

### Model Training Process

**FIGURE 7:**
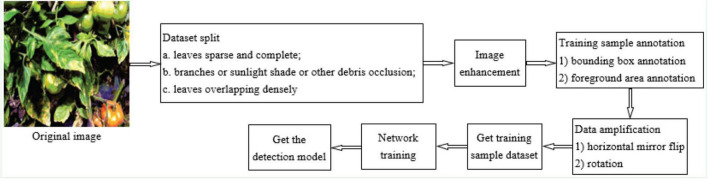
Training flow of tomato diseases and pests object detection network.

The training process of tomato diseases and pests object detection network is shown in Figure 7. After the original image in the training set is equalized by adaptive histogram, the training samples are manually annotated, including bounding box annotation and foreground area annotation; the annotated samples are expanded; and the multiscale training strategy is used for training.

The pseudocode of training YOLOv3-tiny-IRB is shown in Table 6. The number of network layers is *L*, the weight of the network is *W,b*. BN represents batch normalization operation. Also γ and β are the parameters, and they should be updated iteratively in the back propagation process, and λ and α represent momentum value and learning rate, respectively.

**TABLE 6:** The pseudocode of training YOLOv3-tiny-IRB.

Input: Training data
Initialize: *W,b*
for each batch sample X do
for *l*←1 to L do
if *l* = *ConvolutionalLayer* then
$\overset{\sim l}{Z}\leftarrow BN(Z^{l})$
$A^{l}\leftarrow \sigma \left(\overset{\sim l}{Z}+b\right)$
else if *l = Pooling Layer* then
*A*^*l*^←*pool*(*A*^1−1^)
end if
end for
$J\leftarrow \frac{1}{m}\sum_{i=1}^{m}L\left(y^{i},\overset{∼i}{y}\right)$
for *l*←*L* to 1 do
θ*^l^*←{*w*^*l*^, *b*^*l*^, γ*^l^*, β*^l^*}
$V_{d\theta }^{l}\leftarrow \lambda \cdot V_{d\theta }^{l}+(1-\lambda )\frac{\partial J}{\partial \theta^{l}}$
$\theta^{l}\leftarrow \theta^{l}-\alpha \cdot V_{d\theta }^{l}$
end for
end for
Return *W*, *b*, γ, β

**FIGURE 8:**
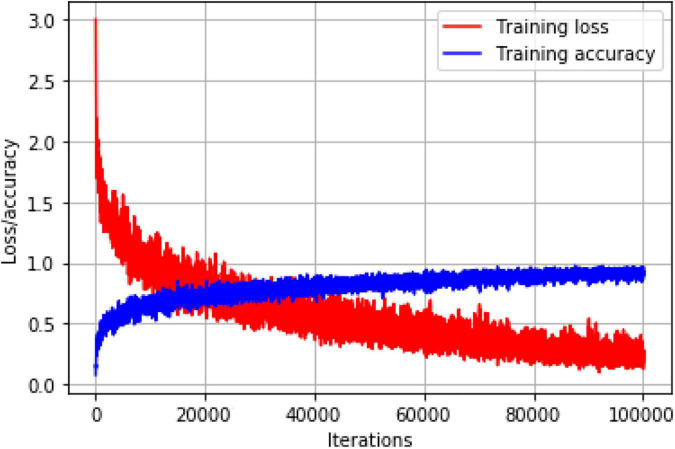
The loss and accuracy curve during training process.

In the training phase, an asynchronous random gradient with a momentum term of 0.9 was used, the initial learning rate of the weights was 0.001, and the attenuation coefficient was set to 0.0005. In view of the differences in object scales of tomato diseases and pests in complex natural scenarios, and since individual object scales are of small size, the network training mainly adopts two strategies. One is to increase the input scale and fine-tune the network at 512 × 512 resolution to adapt to higher input resolution in detection. This strategy can improve the detection accuracy, but also reduce the detection speed. The second strategy is multiscale training. In the training iteration, the network runs every 10 batches from the set multiscale set {384, 416…672} and continue training by replacing one scale randomly again. This strategy makes the model have better detection effect at different input resolution to adapt to multiscale object detection of tomato diseases and pests. The loss descent curve versus the prediction accuracy curve of training set during training process is shown in Figure 8.

According to Figure 8, the accuracy curve is rising steadily whereas the loss curve is decreasing. The accuracy curve gradually, leveled off after 80,000 iterations, and the model at 1,00,000 iterations were selected for this study.

## Experimental Results and Comparative Analysis

The object detection network of tomato pests and diseases was trained with training sets A, B, and C (see Table 1), respectively, and the performance of object detection of tomato diseases and pests in the scene of occlusion and leaf overlap was analyzed, and compared with the performance of the detection models such as Faster RCNN (Ren et al., 2017), YOLO and Adaboost. Around 1,500 images (9,067 tomato diseases and pests objects) were input into the trained network for location regression. When the IOU (intersection and convergence ratio) of the object bounding box predicted by the model and the manually labeled bounding box is more than 0.7, the detection is considered correct, otherwise it is wrong and the test results are obtained.

Precision (P), recall (R), F1 value, and detection speed were selected as evaluation criteria. Sample S is divided into four types according to the combination of the true category of sample S and the predicted category of model: True positive (TP) represents the number of correctly classified positive samples, FP represents the number of incorrectly classified positive samples, false negative (FN) represents the number of incorrectly classified negative samples (FN), and true negative (TN) represents the number of correctly classified negative samples.

Precision (P) represents the proportion of samples that are truly positive in all samples that are predicted to be positive, and the formula is


(6) $$P=\frac{TP}{TP+FP}$$

Recall ^®^ represents the proportion of samples that are predicted to be positive of the truly positive samples. The formula is


(7) $$R=\frac{FP}{FP+TN}$$

The F1 value is a measure function of balancing precision P and recall R, and the calculation formula is


(8) $$F1=\frac{2PR}{P+R}$$

In object detection, in each category the P–R curve can be drawn according to precision P and recall R. The average accuracy AP value of single category detection is the area between P–R curve and coordinate axis, and the calculation formula is as follows:


(9) $$AP=\int_{0}^{1}P\left(R\right)dR$$

The average of AP values of all categories is mAP, and the formula is


(10) $$mAP=\frac{1}{c}\sum AP$$

In the above-mentioned formula C is the number of categories contained in the dataset.

Frame rate per second (FPS) is a common indicator of speed, which is the number of images that can be processed per second.

### Performance Comparison of Several Different Algorithms

The dataset after data processing was used as training set. Deformable Parts Model (DPM) (Sun et al., 2014), Faster R-CNN, Mask R-CNN, single shot multibox detector (SSD) (Liu et al., 2016), YOLOv3, YOLOv3-tiny and YOLOv3-tiny-IRB are taken as a basic network for training and testing respectively.

The test results of different algorithms on the test set are shown in Table 7.

**TABLE 7:** Detection results of different algorithms.

Model name	mAP (%)	F1 score	Detection speed (FPS)
DPM	73.2	0.792	0.3
Faster R-CNN	86.6	0.881	4
Mask R-CNN	87.1	0.889	3.6
SSD	85.3	0.862	55
YOLOv3	88.8	0.897	62
YOLOv3-tiny	88.1	0.893	220
**YOLOv3-tiny-IRB**	**93.1**	**0.922**	**206**

It can be seen that the detection accuracy of YOLOv3-tiny-IRB in this work is much higher than other models. The accuracies of Faster R-CNN, Mask R-CNN, SSD, YOLOv3, YOLOv3-tiny, and YOLOv3-tiny-IRB, which use CNN for convolution feature extraction, are significantly higher than that of DPM algorithm using HOG feature. Traditional object detection algorithm relies on manual designed features, which uses sliding window to select candidate boxes, resulting in severe window redundancy problem and poor generalization performance of feature extraction methods. As a result, the detection accuracy is low and the algorithm steps are numerous, which leads to the slow detection speed and poor real-time performance. Since CNN can simultaneously extract color, texture and shape features, it is superior to traditional methods, and so the performance of CNN detection method is superior.

According to Table 6, the detection speed of DPM detection method in the traditional mainstream machine learning algorithm is the slowest. Faster R-CNN and Mask R-CNN algorithm generates more than 2,000 object candidate region by region, generating a network in the detection process, and then classifies candidate regions by CNN, whereas YOLO series algorithms directly process the whole image by CNN, which reduces the computational complexity, so the detection speed is faster than Faster R-CNN and Mask R-CNN. YOLOv3-tiny is faster than YOLOv3 detection, but there are only two levels of detection and no fusion of small objects, so there is no way to identify objects of different scales well.

Compared with the original YOLOv3 and YOLOv3-tiny, the mAP improved by 4.3 and 5.0%, respectively. The introduction of the inverse-residual module improved the ability of network to extract features and increased the participation of finer feature maps in location regression and classification, thus facilitating the improvement of the detection accuracy of YOLOv3-tiny-IRB. Meanwhile, the inverse-residual block had little effect on the detection speed, and the speed reached 206 frames/s. Therefore, it maintains a good real-time performance while improving the detection accuracy. Overall, YOLOv3-tiny-IRB can achieve trade-off of accuracy and speed, so that the model can be deployed on a large scale in hardware platforms such as embedded devices and mobile terminals to meet the actual needs.

### Detection Results of Amplified Datasets

Based on YOLOv3-tiny-IRB network, the comparison test before and after data amplification was carried out. As can be seen from Table 8, compared with the original image dataset (Training set A), the mAP and F1 score of the model of the enhanced dataset (Training set B) were increased by 2.3 and 0.012%, respectively. After data amplification (Training set C), The mAP and F1 score of the model were improved by 2.8 and 0.021%, respectively compared with the preamplification (Training set A). The results showed that image enhancement, mirror, rotation, and other processing methods could further improve the detection accuracy.

**TABLE 8:** Detection results on different training sets.

Training set	mAP (%)	F1 score
A	90.3	0.901
B	92.6	0.913
**C**	**93.1**	**0.922**

### Detection Results Using Object Foreground Training Samples

To verify the effect of foreground region training samples on detection accuracy, a comparative experiment was conducted. The 3,500 training samples in the original image were divided into two groups: group A training set contained only 21,038 bounding box annotations for all samples, group B contained 2,987 foreground region annotations for occluding and overlapping samples, and 18,051 bounding box annotations for uncovered samples. The training set of group C contains 1,73,304 bounding box annotations, and group D contains 24,158 foreground region annotations, and 1,49,146 bounding box annotations. YOLOv3-tiny-IRB is trained with these four training sets, and the test results on the test set are shown in Table 9. It can be seen that the object foreground region of the tomato diseases and pests training sample is annotated with YOLOv3-tiny-IRB network training. The detection accuracy is obviously improved and the difficulty of occlusion and overlap detection is overcome by reducing the interference of the features of the non-foreground region in the boundary frame. After data amplification, the detection accuracy of the model obtained by using the training set marked by the foreground region is significantly improved compared with the model without foreground region labeling, and the mAP and F1 score of all objects in the test set are improved by 1.4% and 0.024, respectively.

**TABLE 9:** Detection results by training set with foreground region.

Group	Sample numbers	Annotation numbers		mAP (%)	F1 score
		Bounding box	Foreground region		
a	3500	21038	0	88.6	0.899
b	3500	18051	2987	91.7	0.908
c	29016	173304	0	92.8	0.912
**d**	**29016**	**149116**	**24158**	**94.2**	**0.936**

### Detection Results Under Conditions of Occlusion and Overlapping

**FIGURE 9:**
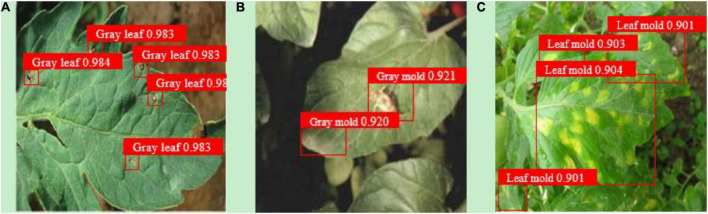
The detection effect diagram of YOLOv3-tiny-IRB [**(A)** deep separation; **(B)** debris occlusion; **(C)** leaves overlapping].

Under the different object detection scenarios of (a) deep separation, (b) debris occlusion, and (c) leaves overlapping, YOLOv3-tiny-IRB trained with dataset C can achieve good detection performance, as shown in Figure 9 and Table 10. It can be seen that the network model designed in this work can detect tomato diseases and pests under a certain degree of occlusion interference and dense overlap of leaves. For the detection of leaves overlapping scenario, YOLOv3-tiny-IRB still reaches 90.2% of mAP, but its detection accuracy is significantly lower than that under deep separation and debris occlusion scenarios.

**TABLE 10:** Detection result comparison.

Object detection scenarios	mAP (%)	F1
(a) Deep separation	98.3	0.971
(b) Debris occlusion	92.1	0.915
(c) Leaves overlapping	90.2	0.901

### Detection Results of Each Species of Diseases and Pests

To discuss detection results of each species of diseases and pests, this study compared the performance of 12 tomato diseases and pests using the improved model. In the original data, there are certain similar symptoms of the disease with similar colors. Pests are easy to discern but are densely distributed, making it more difficult to be fully detected. The detection results of each species of diseases and pests are shown in Table 11.

**TABLE 11:** Detection results of each species of diseases and pests.

Species	Precision (%)	Recall (%)	F1 score
Early blight	93.9	86.5	0.922
Late blight	92.4	85.8	0.901
Gray leaf spot	93.5	86.4	0.912
Brown spot	92.7	84.2	0.910
Coal pollution	93.9	86.1	0.926
Gray mold	94.5	86.9	0.928
Leaf mold	94.8	87.1	0.925
Powdery mildew	92.8	84.3	0.917
Leaf curl	93.2	87.2	0.919
Mosaic	91.1	82.6	0.920
Leaf miner	90.2	82.7	0.904
Greenhouse whitefly	90.1	83.3	0.903
Total	93.9	86.5	0.922

The results showed that the improved model performed excellent in detection accuracy. Detection of twelve different types of diseases and pests all achieved good results, with all F1 scores reaching more than 0.9, and the detection time also reached the requirement of real-time performance. Therefore, the improved model has good generalization ability and can adapt to the needs of rapid detection of tomato pest and disease under natural environmental conditions.

## Conclusion and Future Directions

The experimental results show that the proposed YOLOv3-tiny-IRB algorithm takes into account the simultaneous improvement of detection accuracy and speed, and improves the real-time detection of multiscale objects of occlusion or overlapping tomato diseases and pests in complex natural environment. The research of real-time detection algorithm in complex scenarios can better serve the needs of early warning of plant diseases and pests in smart agriculture. This work not only improves the performance of YOLOv3-tiny network in occlusion or overlapping tomato diseases and pests, but also provides a new method for other object detection, such as fruit harvesting robot, field rabbit, and field bird recognition.

At present, there are many kinds of plant diseases and pests. How to identify more kinds of plant diseases and pests through feature extraction and network structure adjustment and improve the accuracy and efficiency of identification is the direction of follow-up research.

## Data Availability Statement

The original contributions presented in the study are included in the article/supplementary material, further inquiries can be directed to the corresponding author/s.

## Author Contributions

XW designed the research. JL and XW conducted the experiments and data analysis and wrote the manuscript. GL and XW revised the manuscript. All the authors read and approved the manuscript.

## Conflict of Interest

The authors declare that the research was conducted in the absence of any commercial or financial relationships that could be construed as a potential conflict of interest.

## Publisher’s Note

All claims expressed in this article are solely those of the authors and do not necessarily represent those of their affiliated organizations, or those of the publisher, the editors and the reviewers. Any product that may be evaluated in this article, or claim that may be made by its manufacturer, is not guaranteed or endorsed by the publisher.
